# The Socioeconomic Factors of Street Food Vending in Developing Countries and Its Implications for Public Health: A Systematic Review

**DOI:** 10.3390/foods12203774

**Published:** 2023-10-14

**Authors:** Acácio Salamandane, Manuel Malfeito-Ferreira, Luísa Brito

**Affiliations:** Linking Landscape, Environment, Agriculture and Food (LEAF) Research Centre, Associate Laboratory TERRA, Instituto Superior de Agronomia, Universidade de Lisboa, Tapada da Ajuda, 1349-017 Lisbon, Portugal; mmalfeito@isa.ulisboa.pt (M.M.-F.); lbrito@isa.ulisboa.pt (L.B.)

**Keywords:** read-to-eat (RTE) street food, food contamination, hygiene practices, developing countries, environmental sanitation, socioeconomic status, education, public health

## Abstract

The sale of ready-to-eat (RTE) street food represents an important source of income in many developing countries. However, these foods are frequently implicated in outbreaks of gastrointestinal diseases. Street food vendors face several constraints that hamper improvement in the microbiological quality of their products. The aim of this review was to update knowledge about the main causes of foodborne illnesses in developing countries, including the growing concern with the microbial transmission of antibiotic resistance. Following PRISMA guidelines, this systematic review was conducted on original articles published from January 2010 to July 2023. The search was carried out using Scopus, PubMed, Web of Science, Food Science and Technology Abstracts (FSTA), the International Information System for Agricultural Sciences and Technology (AGRIS), as well as isolated searches of relevant articles from Google Scholar. The initial search identified 915 articles, 50 of which were included in this systematic review. The results indicate that, in the majority of the 15 countries examined, women constitute the predominant segment of street food vendors, representing more than 55% of the total number of these vendors. In 11 countries, street food vendors under the age of 18 were identified. Most vendors had a low level of education and, consequently, were unaware of good hygiene practices when handling food. The combination of factors such as poor hygiene practices on the part of food handlers and the lack of facilities, namely, the absence of available potable water, were frequently listed as the main causes of food contamination. Enterobacteriaceae such as *Escherichia coli* (61.9%), *Salmonella* (30.1%), and *Shigella* spp. (9.5%), as well as *Staphylococcus aureus* (30.1%) and *Listeria monocytogenes* (14.3%), were the most common pathogens found in RTE street foods. In 22 studies from 13 developing countries, 59% (13/22) reported high multidrug resistance in Enterobacteriaceae (40% to 86.4% in *E. coli*, 16.7 to 70% in *Salmonella*, and 31 to 76.4% in *S. aureus)*. To address the challenges faced by street vendors and improve their economic activities, it is necessary for government entities, consumers, and vendors to work together collaboratively.

## 1. Introduction

According to the World Health Organization (WHO), approximately 600 million cases of foodborne illness and 420,000 deaths occur each year, with 125,000 deaths among children under five years old, due to unsafe food consumption [1]. This presents a significant burden on public health, economic, and social sectors, particularly in African countries and Southeast Asian regions [2]. Low-income countries are particularly vulnerable to the risk of foodborne illness due to unsafe food preparation; poor individual and collective hygiene; inadequate production; handling, and storage conditions; low levels of literacy and education; and a lack or insufficient implementation of food safety legislation [3,4]. These risks are particularly pronounced in street food vending, which plays a vital socioeconomic role in meeting the nutritional and subsistence needs of low-income urban populations around the world [5].

Ready-to-eat (RTE) foods are pre-cooked, pre-cleaned, and mostly packaged foods that are ready for consumption without prior preparation or cooking [6]. RTE includes salads, cooked meats, smoked fish, desserts, sandwiches, cheese, and food cooked in advance to serve cold, being an increasing commodity in the industrialized world [7]. However, another aspect of these foods is present in developing countries, where RTE street foods are sold by vendors and hawkers in public places for immediate or later consumption without any further processing or preparation [6,8]. Street food is an important source of RTE food in these countries and provides a significant contribution to employment, household income, and affordable food while also being appreciated for its convenience and typical flavors [9,10]. It is estimated that approximately 2.5 billion people consume street food daily. This type of consumption supports the livelihoods of millions of low-income people and contributes significantly to the economy [11]. In developing countries, mainly in Africa, RTE street food vendors are usually associated with small family businesses whose food preparation locations are the vendor’s own homes, sidewalks, or any other informal places [8]. In recent years, in Asian countries such as India, Pakistan, and China, the street food trade has reached new dimensions and is recognized as a phenomenon of great economic and sociocultural importance [12]. In Africa, the contribution of street food vending is largely neglected despite its major contribution to the socio-economic stability of many low-income urban communities [13,14]. It is estimated that in South Africa, in 2022, this informal sector may have been the main source of employment, representing about 18% of citizens working in the street vending sector, where more than 70% of street vendors sold RTE street food [13].

Women and children are the main actors in the street food sector [3,13]. In most cases, these female entrepreneurs are single mothers or widows working in this sector to support the family economy throughout their working lives due to the lack of opportunities in the formal sectors [15,16]. As they are informal sellers, these entrepreneurs have difficulties accessing financing through credit institutions [13,16]. Nor do they count on the support of government entities in their training in good manufacturing practices (GMP) and/or business management [3,15]. These factors make street foods susceptible to microbiological contamination, being an important source of diarrheal disease outbreaks. This type of sale consequently represents a high-risk business for public health [5].

Street food vending is an essential part of urban life in many developing countries. However, the same socioeconomic factors can potentially become a risk to public health. This study aimed to understand how socioeconomic factors of communities, such as income, education, and access to water and solubility facilities, affect the quality and safety of street food. Furthermore, we are deeply interested in comprehending how these dynamics impact public health. Hopefully, this review will provide valuable insights for policymakers and local communities into the need to improve the hygiene conditions of street food vending and, thereby, promote public health in developing countries.

## 2. Methodology

### 2.1. Selection of Relevant Literature

This systematic review was conducted following the recommendations of the Preferred Reporting Items for Systematic Reviews (PRISMA) [17,18]. Briefly, the search terms were defined, and criteria for the inclusion or exclusion of articles to be searched across various databases were established. After conducting the article search, two independent researchers assessed the quality of the selected studies based on the inclusion criteria. In cases of disagreement, a third researcher was consulted to resolve the dispute. Following the selection of articles for inclusion in the study, a thorough analysis was conducted to extract the primary results and conclusions. Subsequently, these data were organized and presented in tables. The databases searched were Scopus, PubMed, Web of Science, Food Science and Technology Abstracts (FSTA), and International Information System for Agricultural Sciences and Technology (AGRIS), in addition to isolated searches for relevant articles found on Google Scholar. A total of 915 articles published from 2010 to July 2023 that mentioned ‘street food vendor’ in the title, keywords, or abstract were analyzed. Over this 13-year span, there was a period of significant change in the food industry, street vending practices, and consumption patterns.

### 2.2. Inclusion and Exclusion Criteria

This review concerns street food vendors or consumers in developing counties and their public health implications. Street foods were considered those prepared at the point of sale or those prepared at home and sold on the street without the existence of a structure built with conventional materials, such as food trailers or mobile kiosks.

This review focused on relevant literature based on combinations of the keywords ‘street food’ OR ‘ready-to-eat street food’ OR ‘street food vendor’ OR ‘street food seller’ OR ‘ready-to eat street food vendor’ OR ‘ready-to-eat street food seller’ AND ‘public health’ OR ‘impact in public health’ OR ‘environment’ OR ‘environmental condition’ OR ‘foodborne’ OR ‘microbial quality’ OR ‘microbial characterization’ OR ‘antibiotic resistance’. In the last chapter of this article, recommendations are presented to improve the quality of street food sold on the street. This was based on the analysis of factors that can contribute to food contamination, namely, knowledge, practices, and attitudes of street food vendors; hygiene and sanitation conditions of the environment; as well as the availability of support infrastructures such as sources of drinking water and toilets.

### 2.3. Screening and Data Extraction

The identified studies were exported to Microsoft Excel to remove duplicates. The selection of articles, conducted by reading titles and abstracts and then the full text, was carried out by two independent researchers, and in case of disagreement, a third researcher was consulted. The studies were systematized by countries, authorship, year, and main conclusions: characterization of RTE street food vendors in terms of knowledge, practices, and attitudes; characterization of the environment and street food vending facilities; assessment of the microbiological quality of food sold on the street; and evaluation of pathogenicity and antibiotic profiles of bacteria isolated from RTE street food.

## 3. Results and Discussion

**Figure 1:**
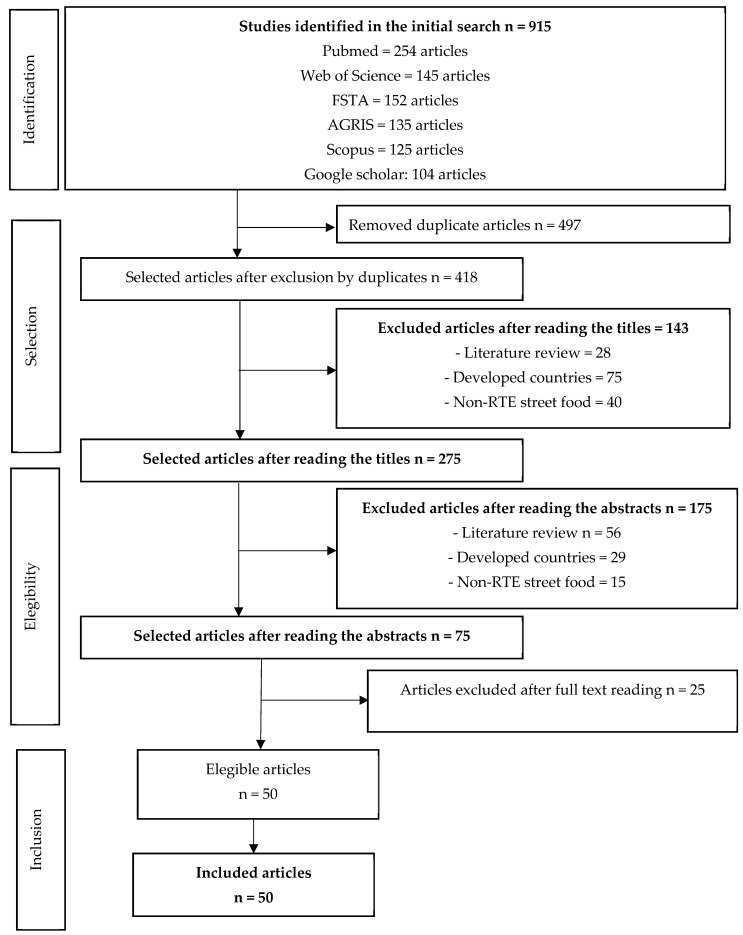
Identification and selection of articles according to PRISMA.

The search strategy identified 915 studies. Four hundred and ninety-seven duplicates were excluded. We excluded 318 studies after screening titles and abstracts. A total of 75 studies remained for full-text screening, after which 25 studies were excluded, leaving 50 studies to be included in this review (Figure 1). Out of the 50 articles included in this study, 22 are dedicated exclusively to evaluating the microbiological quality of street foods and/or detailing the characterization of microorganisms isolated from these foods. Another 14 articles exclusively address the characterization of street food vendors, while 4 articles address hygiene and sanitation conditions. The remaining 10 articles simultaneously explore both the characterization of vendors and the assessment of hygiene and sanitation conditions.

### 3.1. Socio-Economic Characteristics of Street Food Vendors in Developing Countries

In examining the characterization of street food vendors, a comprehensive analysis of 24 studies covering 15 countries, located mainly in Sub-Saharan Africa, was carried out. However, the inclusion also extended to regions of Asia and South America. It is notable that women constitute the predominant segment, representing more than 55% of the total number of street food vendors in all countries examined, except in Haiti and Bangladesh, as indicated in Table 1. Interestingly, the study revealed that, although in smaller proportions, there were cases of underage food vendors identified in 11 of the 15 countries under scrutiny (Table 1). RTE street food vending represents an important business opportunity for many low-income families, as it has a low initial investment cost, combined with the absence of regulation, thus allowing easy entry into the sector [3]. As an alternative to the lack of formal employment, most young people and women turn to the informal sector, mainly street vending [19,20]. Although a considerable number of men are involved in street vending, women are the main vendors, the majority being single or widowed with dependent children [21]. Due to a lack of employment opportunities in the formal sector and a lack of sufficient resources for another type of commercial activity, women resort to selling street food. In addition to this activity requiring a low initial cost, women are the ones who usually prepare the food [3,4,13].

In most countries analyzed, more than half of street food vendors have worked in this sector for more than five years (Table 1), having accumulated a lot of practical experience over time. However, more than 60% of these vendors demonstrate a lack of knowledge about the transmission of foodborne diseases [22], and 60 to 90% of these vendors have never benefited from training on good production and handling practices for RTE foods [22,23,24]. In the context of street food vendor education, in eight of the nine African countries analyzed, more than 35% of vendors had, as their highest educational qualification, a primary level of education (Table 1). This pattern of educational level was also reflected in the results of the study conducted in Malaysia, where it was found that 30.8% of street food vendors had only attended primary school [25].

In some African ethnicities, women do not have the right to education, being often only able to attend school until the primary level. Girls are thus relegated to domestic activities, not having the same opportunities for education and training as boys [12,26]. Girls are trained in food preparation, household chores, and agricultural activities because their main activities are to take care of the house and to prepare for marriage [27]. Premature marriages or unions and educational policies that deny the education of pregnant girls are other factors that contribute to their helplessness in several developing countries, with particular emphasis on Africa [28,29]. All these sociocultural factors contribute to the overall vulnerability of women and make them dependent on men, agriculture, or the informal sector, such as street food sales [30].

According to the World Bank, Sub-Saharan Africa has the lowest adult literacy rate in the world, with less than 60% of its population aged 15 and over able to read and write, which is far below the world rate of 80% [31]. The low level of education makes it difficult to access formal work opportunities. Perhaps it is for this reason that several studies report that most street food vendors have a low level of education [32,33]. As a result of low education, vendors might be unaware of good hygienic practices or of the transmission of pathogenic microorganisms through food and do not show interest in acquiring and implementing new knowledge [11,34].

**Table 1:** Characterization of street food vendors in different developing countries.

Region	Countries	References	Predominance of						
			Woman	Age (≥18 Years Old)	Up to Primary School	Secondary or High School+	Single Status *	Absence of Training	Work Experience **
Africa	Mozambique	Salamandane et al. (2021) [3]	79.5%	73.9%	58.9%	41.1%	82.8%	83.6%	54.1%
	Kenya	Muendo et al. (2022) [24]	77.5%	100%	31.5%	68.2%	48.3%	90.1%	66.9%
	Uganda	Muyanja et al. (2011) [35]	87.6%	93.3%	70.8%	29.2%	43.6%	-	-
	Cameroon	Maffouo et al. (2021) [36]	100%	100%	3.3%	96.7%	-	-	-
	Ethiopia	Werkneh et al. (2023) [37]; Adane et al. (2018) [38]; Azanaw et al. (2022) [39]	74.4–83%	85–92.7%	49.2–55%	45–50.8%	60–76%	65.6%	5–21%
	Nigeria	Okojie et al. (2014) [23]	90.2%	-	50.3%	49.7%	-	71.3%	-
	Tanzania	Basheikh et al. (2023) [40]	91.4%	100%	70.8%	29.2%	87%	-	26.8%
	South Africa	Marutha et al. (2020) [41]; Mahopo et al. (2022) [13]; Nkosi et al. (2021) [42]	59.6–90.2%	97.4–100%	37.8–65.3%	62.1–34.5%	69.3–72%	79.9%	41.7–90%
	Ghana	Danikuu et al. (2015) [43]; Tuglo et al. (2021) [44]	76.7–83.5%	95.3%	47.9%	52.1%	23.6%	56–100%	-
Asia	Indonesia	Putri et al. (2021) [22]	70.8%	-	65.2%	34.8%	-	63.9%	-
	Vietnam	Huynh-Van et al. (2022) [30]; Samapundo et al. (2015) [45]	73.8%	93.7%	8.3–20.4%	79.6–91.7%	-	68.2–95%	37.5%
	Bangladesh	Hossen et al. (2021) [46]; Kundu et al. (2021) [47]; Meher et al. (2022) [48]	4–7.3%	98.5–100%	59.1–74.5%	25.5–40.9%	8.5–16.2%	100%	70–77%
	Malaysia	Jores et al. (2018) [25]	59.8%	-	30.8%	69.2%	20.5%	64.1%	-
Latin America	Haiti	Samapundo et al. (2015) [49]	37.5%	65.3%	26.8%	73.2%	-	88.7%	-
	Brazil	Da Silva et al. (2014) [50]; da Vitória [51]	55.9–96.5%	100%	40.7–48.7%	51.3–59.3%	53%	-	2–60.5%

* Including widowed; ** More than 5 years in street food vending.

### 3.2. Environmental Conditions and Sanitary Facilities

The safety of RTE street food is affected by the quality of the raw materials used. Microbial cross-contamination of food can occur along the entire production chain, from processing, transport, storage, display, and preparation to serving food for consumption [1]. The combination of factors such as poor hygiene practices by food handlers and the lack of facilities, namely, a lack of potable water; inadequate infrastructure; food storage at temperatures that favor microbial growth; and exposure of food to animals, including rodents and insects, are often listed as major problems [1,38,44,48]. Fresh ingredients such as fish, meat, and fresh vegetables used in food preparation are also purchased in informal markets, which, in most cases, do not have adequate conditions for selling these products [4,52]. Cases of a lack of sectorization of these informal markets are frequently reported, in which it is possible to find domestic animals for slaughter, mainly chickens and goats, for sale alongside the sale of fish and meat or even fresh vegetables [4,53]. These practices are encouraged by the lack of regulation and inspection in these countries.

Salamandane et al. (2021) [3] analyzed the environment of street food vendors in Mozambique and observed that most vendors did not have access to potable water and toilets at the cooking and/or selling place (Table 2). Remaining in Mozambique, most municipal markets, essential sources of raw ingredients used to prepare RTE street food, such as fresh vegetables, fruits, and meat, do not have access to potable water [4]. Additionally, sanitary facilities in these markets are in a significantly dilapidated state [3,4]. This situation contributes to product contamination. Indeed, the scarcity of potable water sources in places where street food is prepared and/or sold seems to be prevalent in most developing countries. Studies conducted in Lesotho [54] revealed that over 90% of street food vendors did not have access to tap water, and 40% of vendors did not have access to toilet facilities (Table 2). However, even under these conditions, 84% of these vendors used reusable plates that were ‘cleaned’ after each use [54].

In Uganda, a high percentage of coverage of access to potable water was observed among street food vendors. In fact, more than 90% had access to tap water at the point of preparation and/or sale of street food. However, more than 80% stated that they did not have access to a clean toilet and used reusable plates [35]. The availability of potable water was also investigated in studies conducted in Brazil and Cameron, with coverage exceeding 76%, and in Vietnam, with coverage exceeding 67% of street food vendors [30,36,50].

As evidenced in Table 2, a significant number of street food vendors opt for reusable plates, underlining their commitment to environmental sustainability. However, due to the lack of facilities, mainly the lack of tap water and dishwashing facilities, these utensils are usually washed in a basin with standing water, which prevents them from being cleaned correctly. Poorly washed utensils can serve as a vector for transmitting pathogens from a sick individual or asymptomatic carrier to a healthy individual [3,55]. In this case, not only foodborne pathogens can be transmitted, but also other pathogens can be transmitted through contact with human fluids, such as saliva. *Mycobacterium tuberculosis* is one such pathogen that causes tuberculosis, as well as agents of viral diseases such as *herpes labialis* [56]. In many developing countries, particularly within sub-Saharan Africa, lack of access to clean water is playing a significant role in the increase in food and waterborne diseases. Several countries in this area depend on sporadic water supply systems, where consumers have access to piped water for significantly less than 24 h a day [57]. The susceptibility to waterborne diseases resulting from microbial contamination in these intermittent supply setups remains remarkably high. This is mainly attributed to the infiltration of pathogens into non-pressurized or low-pressurized pipelines through mechanisms such as intrusion, backflow, particle release, or biofilm detachment [57,58,59].

**Table 2:** Percentage of street food vendors with access to facilities in developing countries.

Region	Countries	Reference	Potable Water (%)		Toilet Access (%)		Utensils Re-Used (%)	
			Yes	No	Yes	No	Yes	No
Africa	Mozambique	Salamandane et al. (2021) [3]	34.4	65.6	9.3	90.7	100	-
	Kenya	Kariuki et al. (2017) [60]	53.1	46.9	46.3	53.7	42.9	57.1
	Lesotho	Letuka et al. (2021) [54]	2	98	60	40	84	16
	Uganda	Muyanja et al. (2011) [35]	99.1	0.9	16.9	83.1	87.1	12.9
	Cameron	Maffouo et al. (2021) [36]	76.7	23.3	-	-	100	-
	Ethiopia	Werkneh et al. (2023) [37]	18.3	81.7	-	-	90.6	9.4
		Negassa et al. (2023) [61]	12.3	87.7	48.2	51.8	100	-
	Nigeria	Murutha et al. (2020) [41]	29.2	69.8	64.7	35.3	88	22
	South Africa	Nkosi et al. (2021) [42]	27.5	72.5	-	-	41.5	58.5
Asisa	Vietnam	Huynh-Van et al. (2022) [30]	67.4	32.6	-	-	-	-
	Malaysia	Jores et al. (2018) [25]	33.3	67.7	-	-	38.5	61.5
	Bangladesh	Kundu et al. (2021) [47]	18.5	81.5	-	-	65.7	34.3
		Khairuzzaman et al. (2014) [62]	47	53	15	85	48	52
Latin America	Haiti	Samapundo et al. (2015) [49]	35	65	-	-	100	-
	Brazil	Da Silva et al. (2014) [50]	76.9	23.1	-	-	-	-

### 3.3. Consumer Safety Awareness and Education

The perception of street food quality results from the comparison of customer expectations and perceived performance [63]. This perception is based on the type of service, including the organization of the point of sale, cleanliness, and consumer safety (in terms of health), among other aspects [63,64]. However, cultural factors such as the preference for traditional foods, purchasing power, and consumer knowledge are decisive for the quality and safety of RTE food sold on the street [64]. Since food safety is an attribute that is often not possible to be observed, it is not valued by consumers, tending to be clearly insufficient in non- or poorly regulated markets.

According to Samapundo et al. (2015) [45], most street food consumers in Ho Chi Minh City, Vietnam, do not have good knowledge about food safety and do not associate food safety with possible outbreaks of diarrheal diseases. In a study conducted in Mozambique, Salamandane et al. (2020) reported that women demonstrated greater knowledge about food safety and a higher level of use of basic hygiene rules when compared to men. A study in Haiti reports that street food vendors were determined to have higher levels of food safety knowledge than consumers. Most consumers were aware of the importance of hand washing and proper cleaning when it comes to preventing foodborne diseases but had difficulty identifying groups at risk for foodborne diseases [49]. A study conducted in Handan, China [11], concluded that younger consumer’s attitudes toward food safety were significantly better than those of older age groups. This difference was correlated with the educational level and, correspondingly, with the relatively high income of younger individuals [11].

In fact, a lack of consumer education and awareness is a common problem in low- and middle-income countries, affecting food safety and sustainability [65]. The implementation of consumer awareness will represent a change in demand for safer and more sustainable food. This shift in consumer choices and behavior requires time and a targeted approach. Education to raise awareness about the importance of food safety and dietary standards is one way to empower consumers. Education gives consumers the tools to raise their voices and increase the demand for quality and safer products and services on the market [65,66].

### 3.4. Public Health Implications of Street Food Vending

#### 3.4.1. Main Foodborne Pathogens

Street food vending is a social, economic, and cultural activity in an evolving world. This type of activity contributes to providing accessible and low-cost meals to urban populations, thus ensuring food and nutritional security for this population throughout the world, particularly in lower-income countries [1]. Additionally, street foods attract tourists as they are convenient, relatively inexpensive, and offer unique flavors and experiences that can reflect traditional local cultures. Despite the strong contribution of street food to improving the income of many low-income families in developing countries, street food is often associated with outbreaks of food- and -water-borne diarrheal diseases [67].

Enterobacteriaceae (*Escherichia coli*, *Salmonella,* and *Shigella* spp.), *Staphylococcus aureus,* and *Listeria monocytogenes* are the most common pathogens found in RTE street foods (Table 3). Pathogenic strains of *E. coli* have been found in several RTE street foods in Nigeria [68,69], Mozambique [3,70], Ethiopia [70], and Egypt [71]. Contamination with *E. coli* O157:H7, the most common pathotype causing enterohemorrhagic disease (EHEC), possessing the *stx* gene that encodes Shiga toxin, has been frequently reported (Table 3). In addition to this pathotype, contamination of RTE street foods has been reported with other pathotypes, such as enterotoxigenic (ETEC), enteropathogenic (EPEC), enteroinvasive (EIEC), and enteroaggregative (EAEC) [68,70]. *Salmonella* and *Shigella* are other foodborne gastrointestinal pathogens linked to the consumption of RTE street food. In Pakistan, *Salmonella* Typhimurium and *Salmonella* Enteritidis serovars were found in 38% of analyzed RTE street foods [72]. *Salmonella* Typhimurium has also been reported in other countries, such as Bangladesh and Nigeria (Table 3), as one of the most important foodborne pathogens. The quality of water consumed in most developing countries also represents an important source of diarrheal diseases [58].

Foodborne outbreaks of *L. monocytogenes* and *S. aureus* (Table 3) are often associated with the consumption of RTE street foods. Recently (from January 2017 to July 2018), a foodborne listeriosis outbreak was reported in South Africa, with 1060 laboratory-confirmed cases of listeriosis and 216 deaths recorded [73,74]. Epidemiological investigations revealed that this outbreak was related to cross-contamination with *L. monocytogenes* in the facility plant of a major producer of RTE processed meat [73]. *L. monocytogenes* was detected in RTE street food sold in Nabia and India (Table 3). In India, contamination with *L. monocytogenes* has been associated with RTE street foods produced with milk or dairy products and fruit, such as curds, cheese, banana milkshakes, chocolate, almond milk, and strawberries [75]. Meanwhile, in Namibia, the contamination was related to RTE meat sold on the street [76].

The growth in food matrices of certain strains of *Staphylococcus*, particularly *S. aureus*, can lead to the production of enterotoxins. These enterotoxins have the potential to induce staphylococcal food poisoning, which is commonly characterized by severe nausea and vomiting that occurs within a period of 2 to 8 h after consumption of contaminated food. It is important to highlight that the cooking process effectively eliminates *S. aureus*, but it is insufficient in deactivating the heat-stable enterotoxins produced by these bacteria [77]. *S. aureus* was identified in RTE street foods in 8 of the 13 countries examined in this study (Table 3). In *Staphylococcus* recovered from RTE street foods sold in Mozambique, genes (*seA*, *seC,* and *seD*) coding for staphylococcal enterotoxins were detected [78]. Also in Benin, genes coding for staphylococcal enterotoxins (*seA*, *seB,* and *seD*); TSST-1; and toxins ETA, PVL, and LukE/D were found in *S. aureus* isolated from RTE street food [79]. *S. aureus* is among the normal microbiota of the skin and upper respiratory tract of healthy humans and can be transmitted to RTE street food, mainly through asymptomatic food handlers [80].

Furthermore, other enteric pathogens, such as *Vibrio cholerae*, *Cryptosporidium,* and *Giardia lamblia*, are frequently reported in waterborne and foodborne outbreaks in many Sub-Saharan African countries [81,82].

**Table 3:** List of the most important foodborne pathogenic bacteria reported in several developing countries and their antibiotic resistance profile.

Region	Countries	Reference	Bacteria	Serovar/Virulence Gene or Factor	Most Prevalent Antibiotic Resistance	
					Phenotype	Genotype
Africa	Egypt	Khalil et al. (2016) [71]	*E. coli*	*stx1*, *stx2*, *eaeA*, and *hlyA*	Multidrug resistance (84%)	-
		El-Shenawy et al. (2011) [83]	*Listeria* spp.	*L. monocytogenes*	-	-
	Namibia	Shiningeni et al. (2019) [76]	*E. coli*, *S. aureus*, *L. monocytogenes,* and *Shigella*	-	-	-
	Mozambique	Salamandane et al. (2022) [70]	*E. coli*	*stx*, EAST1, *eaeA*, *lt,* and *st*	Imipenem (35.5%), Tetracycline (58%)	*bla_TEM_*, *bla_SHV_*, *blaCTX-M*, *ACC*, *FOX*, *tet,* and *sul*
		Salamandane et al. (2022) [78]	*Staphylococcus* spp.	*hlb*, *seA*, *seC*, *seD,* and *sak*	Multidrug resistance (59.6%)	*bla-Z*, *mecA*, *vancA*, *ermA-C*
	Benin	Sina et al. (2011) [79]	*S. aureus*	*seA*, *seB*, *seD*, TSST-1, ETA, PVL, and LukE/D	Penicillin (100%), Erythromycin (80%)	-
	Burkina Faso	Nikiema et al. (2021) [84]	*Salmonella*	*S*. Enteritidis	Multidrug resistance (16.7%)	*bla_TEM_*, *catA. tetA*, *gyrA S83F* and *D87Y; parC S80I*
	Ghana	Aduah et al. (2021) [85]	*Salmonella*	-	Azithromycin (83.3%), Teicoplanin (100%)	-
		Ayamah et al. (2021) [86]	*S. aureus* and *E. coli*	-	Multidrug resistance (51.85% of *E. coli*, 69.44% *of S. aureus*)	-
		Karikari et al. (2022) [87]	*E. coli* and *Salmonella*	-	Cefotaxime, ampicillin, Augmentin, tetracycline, cefepime, trimethoprim/sulfamethoxazole, ceftriaxone (50–100%)	ESBL production (55.4% of *E. coli* and 44.6% of *Salmonella*)
	Ethiopia	Alelign et al. (2023) [88]	*E. coli*, *Salmonella*, *S. aureus*	*E. coli* O157:H7	Multidrug resistance (31% of *S. aureus*, 33.3% of *Salmonella,* and 40% of *E. coli*)	-
		Eromo et al. (2016) [89]		-	Ampicillin (100%), Chloramphenicol (33.8%)	-
	Nigeria	Beshiru et al. (2022) [68]	*E. coli*	ETEC, EPEC, EAEC, EIEC, and EHEC	Multidrug resistance (77.4%)	-
		Anab-Atulomah et al. (2021) [69]	*E. coli*, *Salmonella*, *S. aureus,* and *Klebsiella pneumoniae*	*Salmonella* Typhimurium	Multidrug resistance (40% of *S. aureus*)	ESBL production (36% of *E. coli* and 17% of *Salmonella*)
	Algeria	Yaici et al. (2017) [90]	*E. coli*, *K. pneumoniae*	-	Multidrug resistance (65.5%)	*blaCTX*, *blaSHV*, *blaDHA*, *blaCMY,* and *aac(6′)-Ib-cr*
Asia	Bangladesh	Ema et al. (2022) [91]	*E. coli*	-	Multidrug resistance (47.6%)	-
		Nahidul-Islam (2022) [92]	*E. coli*, *Salmonella*, *S. aureus,* and *Klebsiella pneumoniae*	*Salmonella* Typhimurium	Ciprofloxacin, Tetracyclin, Fosfomycin	-
	Pakistan	Raza et al. (2021) [72]	*Salmonella*	*S.* Typhimurium; *S.* Enteritidis	Amoxicillin (90%), Erythromycin and Chloramphenicol (100%)	-
	Nepal	Kunwor et al. (2022) [93]	*E. coli*, *S. aureus*, *Salmonella, and Shigella*	-	Multidrug resistance (100% of *Shigella*, 76.4% of *S. aureus*, 70% of *Salmonella*, 69.38% of *E. coli*)	-
		Adhikari et al. (2023) [94]		*E. coli* O157:H7	Multidrug resistance (65.85% of *E. coli*, 45.16% of *Salmonella*)	*bla*CTX-M, *bla*VIM
	India	Elavarasi et al. (2023) [75]	*L. monocytogenes*	-	Methicillin (52%), Teicoplanin (56%)	
		Giri et al. (2021) [95]	*E. coli* and *K. pneumoniae*	-	Multidrug resistance (86.44%)	*blaCTX-M*, *bla_TEM_*, *blaSHV,* and *blaNDM*

#### 3.4.2. Transmission of Antibiotic Resistance Genes

In addition to the direct effect of pathogens on foodborne diseases, there is another risk, which is the transmission of antibiotic-resistance genes from these to other bacteria. Antibiotic-resistant infections are on the rise, both in the general population and in healthcare settings, posing a significant public health challenge to already overburdened healthcare systems [96]. Due to the overuse and misuse of antibiotics in humans and livestock, bacteria are becoming increasingly resistant to commonly used treatments, making infections difficult, and sometimes impossible, to cure [97]. This has led to an increase in the burden of hospitalizations and deaths with very high costs for society [96,97]. Potential health risks from antibiotic resistance have been linked to farm-to-table contamination of food by strains of *E. coli*, *Salmonella* Typhimurium, Shigella, *S. aureus,* and other antibiotic-resistant foodborne species [89,98].

Table 3 summarizes the antibiotic resistance profile (phenotype and genotype) of bacteria recovered from RTE street food, corresponding to 22 studies from 13 developing countries. A total of 59% (13/22) of studies reported high multidrug resistance in Enterobacteriaceae, ranging from 40% to 86.4% in *E. coli*, from 16.7 to 70% in *Salmonella*, and from 31 to 76.4% in *S. aureus* (Table 3). In Nigeria, 36% of *E. coli* and 17% of *Salmonella* isolated from RTE street food were ESBL producers [69].

Zurfluh et al. (2015) [99] found 78.3% multidrug-resistant and extended-spectrum β-lactamase (ESBL)-producing Enterobacteriaceae in RTE salads, fresh-cut fruit, and sprouts imported from the Dominican Republic, India, Thailand, and Vietnam. ESBLs are enzymes that hydrolyze penicillin, as well as aztreonam and first-, second-, and third-generation cephalosporins [87,100]. The problem of ESBL-producing bacteria has been identified in medical systems and communities worldwide [100,101]. One of the main reasons for this rapid spread is that ESBL-producing bacteria can be transmitted through contaminated food or water [102,103].

In Mozambique, multidrug resistance was related to the presence of β-lactamase resistance genes, namely, the *bla_TEM_*, *bla_SHV_*, *bla_CTX-M_*, ACC, FOX, tetracycline, and sulfonamide resistance genes [78]. In India [95], Burkina Faso [84], and Algeria [90], β-lactamase genes have also been associated with antibiotic resistance in enterobacteria recovered from RTE street food.

*S. aureus* is an important foodborne pathogen due to the combination of toxin-mediated virulence, invasiveness, and antibiotic resistance. In RTE street food sold in Mozambique, Salamandane et al. (2022) [78] found 59.6% of multidrug-resistant staphylococci with a high level of resistance to methicillin and vancomycin. In Gana, Ayamah et al. (2021) [86] found 69.44% multidrug resistance in *S. aureus* recovered from RTE street food (Table 3). In Ethiopia, 31% of *S. aureus* recovered from RTE street food were resistant to methicillin [88].

Infections with methicillin-resistant strains of *S. aureus* (MRSA) have been linked to hospital-acquired infections (HA-MRSA) [78,104]. However, in recent decades, there has been an increase in the number of community-acquired (CA-MRSA) and livestock-associated (LA-MRSA) MRSA infections [105,106]. Some authors associate this increase in CA-MRSA and LA-MRSA infections with RTE food contamination, either through contaminated raw materials or through contamination by food handlers [78,98].

## 4. How to Change the Situation

As mentioned before, street food vendors are often poor, less educated, and lack knowledge about safe food handling. Furthermore, there are problems inherent to the environment in which food is prepared and/or sold, including, in most cases, the absence of potable water. In fact, this problem has been tackled under the framework of WHO’s Five Keys to Safer Food to assess and promote food safety (keeping clean, separating raw and cooked foods, cooking thoroughly, keeping food at safe temperatures, and using safe water and raw materials [10].

**Figure 2:**
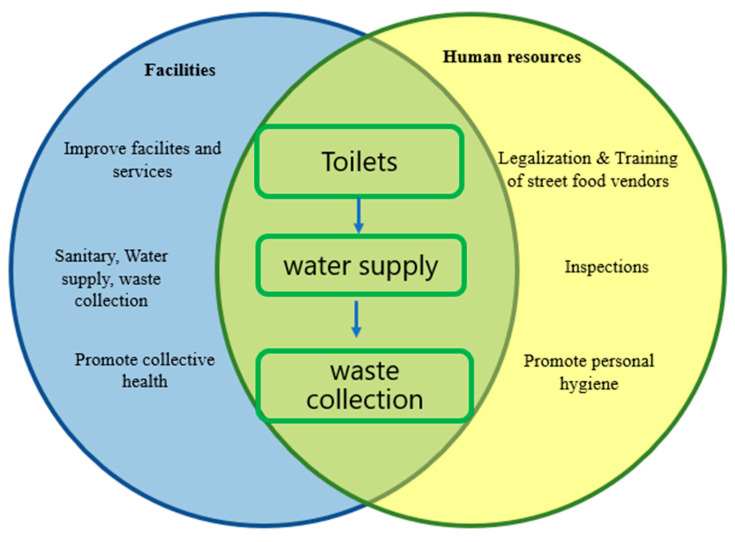
Primary actions to promote the quality and microbiological safety of street foods.

However, negligent behavior by vendors, including the use of contaminated raw food and ingredients, inadequate food storage, lack of personal hygiene during food preparation, inadequate cooling and reheating of foods, and a prolonged time lag between food preparation and consumption, continue to be commonly observed [60,107]. To change this situation, concerted measures are needed, involving all stakeholders in society, from law enforcement entities to educational programs for vendors, consumers, and the general population (Figure 2). The fact that, despite their poor knowledge, vendors have positive attitudes towards food safety [20] is an opportunity to launch appropriate educational programs. In fact, it was found that the acquisition of good food-handling practices is associated with the training of vendors and not their educational level [108].

### 4.1. Legalization and Training of Street Food Vendors

In low-income countries, street vendors often work informally and are not recognized by governmental institutions. Instead of legalizing them, successive municipal governments persecute them, and they are often looted and arrested by police authorities [109]. By not legalizing them, local governments ignore the existence of structural problems in the local economy that cause these cases to drag on for a long time while vendors gain space. The legalization of street food vendors is a condition for the organization of the sector. This legalization would facilitate the provision of short-term training courses in good food processing and handling practices and in business management and entrepreneurship [13].

Concerning business, street vendors have few managerial skills. However, street food vending contributes to the local economy, providing employment for many families. Therefore, in addition to training in good food production and handling practices, there is also a need to provide financial resources and small business management skills [110]. Both training in food safety and business management would be drivers of change that would make street food vendors begin to have more proactive attitudes.

### 4.2. Appropriate Legislation and Intensification of Inspections

Food safety inspection allows continuous assessment of the quality of food products, as well as the methods, equipment, and environment where food is stored and exposed for public consumption [111]. This process aims to minimize the risk of food contamination by ensuring that food is processed properly and in a sanitary environment. In developed countries, inspection of the food safety system occurs at various points in the supply chain, from the production area to the final consumer. However, in most developing countries, food safety systems are weak, fragmented, and not effective in promoting or protecting consumer health [112]. Monitoring street food vendors is a difficult and sometimes impractical task, as they are not registered and many of them do not have fixed points of sale [3,4]. For this reason, producing laws and regulations that allow street food vendors to work legally will create conditions for local governments to oversee this activity and, in some cases, control the quality of food throughout the supply chain. However, before governments think about inspection activities, they need to guarantee basic conditions, such as water supply infrastructure, sewage, and sanitation systems (Figure 2). Together with the education of vendors, these are undoubtedly the main challenges in ensuring the safety of street foods [53]. Improving the food safety system requires an investment that many countries do not have or are not willing to pay. On the other hand, due to the prevalence of high levels of malnutrition and the lack of sufficient food for the majority of the population, the concept of food security overlaps with the concept of food safety [113]. Therefore, these countries face a real challenge in producing and enforcing food safety legislation without compromising food availability [112].

### 4.3. Improvement in Environmental Sanitation and the Water Supply Network

Lack of proper sanitation and waste disposal facilities are among the factors that contribute to street food contamination by vendors [60]. To attract consumers, most points of sale are located near busy places such as bus stations, markets, schools, and clinics or hospitals [3,60]. These places do not have basic facilities such as toilets, running water, or properly conditioned garbage containers. Together with insufficient vendor knowledge and negligent behavior, these are considered the most relevant factors contributing to foodborne disease outbreaks [49,114].

Food quality assurance is strongly influenced by water quality [115]. As these foods are, in some cases, produced at the point of sale, improving the quality and quantity of the water supply would undoubtedly reduce the risk of contamination of RTE street foods (Figure 2). The water supply contributes to improving individual and collective hygiene and guarantees the correct washing of utensils used in the preparation, sale, and consumption of these foods [60,115].

Market cleanliness and solid waste (garbage) management are other factors that can be improved to guarantee food quality and safety. The presence of garbage near food production, sale, and consumption areas attracts insects and other disease vectors, mainly flies, rats, and cockroaches [3], which can cause diseases such as cholera and malaria. In addition, these diseases affect the most vulnerable populations since, as already mentioned, women are the most common gender in these types of markets and tend to take their children to this work, ignoring their higher susceptibility to these diseases.

### 4.4. Implementation of Public Health and Sanitary Education Policies

The successful implementation of street food vending policies that align with public health policies remains a significant challenge in many developing countries due to several factors [116,117]. First, compliance with licensing requirements, vendor training, and infrastructure arrangements for policy implementation are necessary but demanding. Secondly, the demand for safe food requires the introduction of regulations focused exclusively on food safety and hygiene, in addition to monitoring nutritional quality [117,118]. While investing in additional infrastructure requires significant financial and human resources, adopting a healthy food sales policy offers the potential for new business and employment opportunities, generating tax revenue and reducing the costs of treating foodborne illnesses [116,119].

Education, both in general and specifically focused on sanitation, can be an effective tool to promote food safety and achieve positive outcomes in the medium and long term. Providing food hygiene instruction during primary school is essential, as behavior is more malleable at this stage, making it easier to influence habits and establish a solid foundation for lifelong food safety practices [120]. Numerous countries prioritize public education and information about food safety as a critical method for mitigating foodborne illnesses. The impact of an informed and educated public goes beyond concerns regarding the safety of RTE products. It often shapes public policies regarding food quality and industry practices [121].

## 5. Study Limitations

One of the limitations encountered in this study is the absence of readily available data on street food vending in most developing countries. Many developing nations lack detailed records on street food vending, making it challenging to obtain a comprehensive picture. In many of these countries, the information published in scientific articles tends to focus on specific geographic areas, such as capital cities and major metropolitan regions. In other cases, data are provided in non-peer-reviewed or unindexed journals, reducing the comprehensiveness and reliability of the data and further complicating the attainment of a complete and accurate understanding of the situation.

On the other hand, the heterogeneity of contexts in developing countries is a critical consideration. Economic, social, and cultural differences among regions can make it difficult to generalize results from one area to another.

## 6. Conclusions

The contribution of street food to the economic livelihoods of low-income families justifies maintaining a continued focus on improving food safety. In this systematic review, the challenges of street food vending in developing countries were discussed, regarding its importance to low-income families, with a special focus on women, single mothers, and widows. We observed that the presence of potentially pathogenic microorganisms in RTE street foods is associated with both the lack of hygienic conditions and food storage suitability at the point of preparation and/or sales, as well as the generally low level of education and low food safety literacy observed among most vendors in various developing countries. To address the challenges faced by street food vendors and improve their economic activities, it is necessary for government entities, consumers, and vendors to work together collaboratively to promote training programs aimed at providing stakeholders with the knowledge, values, attitudes, commitments, and skills needed to produce and sell safe food and protect and improve the environment in which they operate.
